# 7′-Methyl-5′-oxo-2′,3′-dihydro­spiro­[1,3-dioxolane-2,1′(5′*H*)-indolizine]-6′-carbonitrile

**DOI:** 10.1107/S160053681003285X

**Published:** 2010-08-28

**Authors:** Kai Wang, Wen-ge Yang, Lu-Lu Wang, Jing Zhu, Yong-hong Hu

**Affiliations:** a State Key Laboratory of Materials-Oriented Chemical Engineering, School of Pharmaceutical Sciences, Nanjing University of Technology, Xinmofan Road No. 5 Nanjing, Nanjing 210009, People’s Republic of China; b State Key Laboratory of Materials-Oriented Chemical Engineering, College of Life Science and Pharmaceutical Engineering, Nanjing University of Technology, Xinmofan Road No. 5 Nanjing, Nanjing 210009, People’s Republic of China

## Abstract

In the title compound, C_12_H_12_N_2_O_3_, the five-membered ring attached to the aromatic ring adopts an envelope conformation with a C atom in the flap position. The spiro-linked five-membered ring adopts a twisted conformation. In the crystal, C—H⋯O hydrogen bonds link the mol­ecules into *C*(5) chains propagating in [001].

## Related literature

For medicinal background, see: Takimoto & Calvo (2008 ▶). For further synthetic details, see: Wani *et al.* (1980 ▶). For bond-length data, see: Allen *et al.* (1987 ▶).
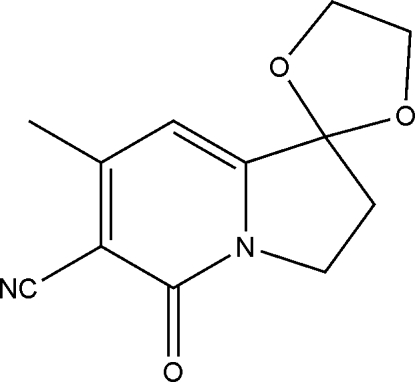


## Experimental

### 

#### Crystal data

C_12_H_12_N_2_O_3_
*M* *_r_* = 232.24
Orthorhombic, 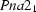

*a* = 7.9460 (16) Å
*b* = 25.945 (5) Å
*c* = 5.3430 (11) Å
*V* = 1101.5 (4) Å^3^
*Z* = 4
Mo *K*α radiation
μ = 0.10 mm^−1^
*T* = 293 K
0.30 × 0.10 × 0.10 mm

#### Data collection

Enraf–Nonius CAD-4 diffractometer
Absorption correction: ψ scan (North *et al.*, 1968 ▶) *T*_min_ = 0.970, *T*_max_ = 0.990
2217 measured reflections
1123 independent reflections
821 reflections with *I* > 2σ(*I*)
*R*_int_ = 0.035
3 standard reflections every 200 reflections  intensity decay: 1%

#### Refinement

*R*[*F* ^2^ > 2σ(*F* ^2^)] = 0.046
*wR*(*F* ^2^) = 0.112
*S* = 1.00
1123 reflections
155 parameters
1 restraint
H-atom parameters constrained
Δρ_max_ = 0.20 e Å^−3^
Δρ_min_ = −0.20 e Å^−3^

### 

Data collection: *CAD-4 EXPRESS* (Enraf–Nonius, 1994 ▶); cell refinement: *CAD-4 EXPRESS*; data reduction: *XCAD4* (Harms & Wocadlo, 1995 ▶); program(s) used to solve structure: *SHELXS97* (Sheldrick, 2008 ▶); program(s) used to refine structure: *SHELXL97* (Sheldrick, 2008 ▶); molecular graphics: *SHELXTL* (Sheldrick, 2008 ▶); software used to prepare material for publication: *PLATON* (Spek, 2009 ▶).

## Supplementary Material

Crystal structure: contains datablocks global, I. DOI: 10.1107/S160053681003285X/hb5573sup1.cif

Structure factors: contains datablocks I. DOI: 10.1107/S160053681003285X/hb5573Isup2.hkl

Additional supplementary materials:  crystallographic information; 3D view; checkCIF report

## Figures and Tables

**Table 1:** Hydrogen-bond geometry (Å, °)

*D*—H⋯*A*	*D*—H	H⋯*A*	*D*⋯*A*	*D*—H⋯*A*
C5—H5*B*⋯O3^i^	0.97	2.49	3.275 (5)	138

Symmetry code: (i) 
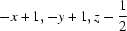
.

**Fig. 1.**
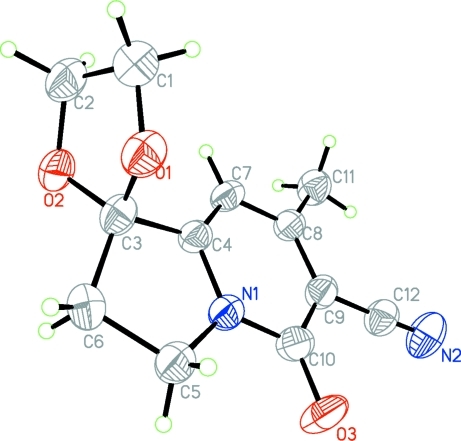
The molecular structure of (I) with displacement ellipsoids are drawn at 30% probability levels.

**Fig. 2.**
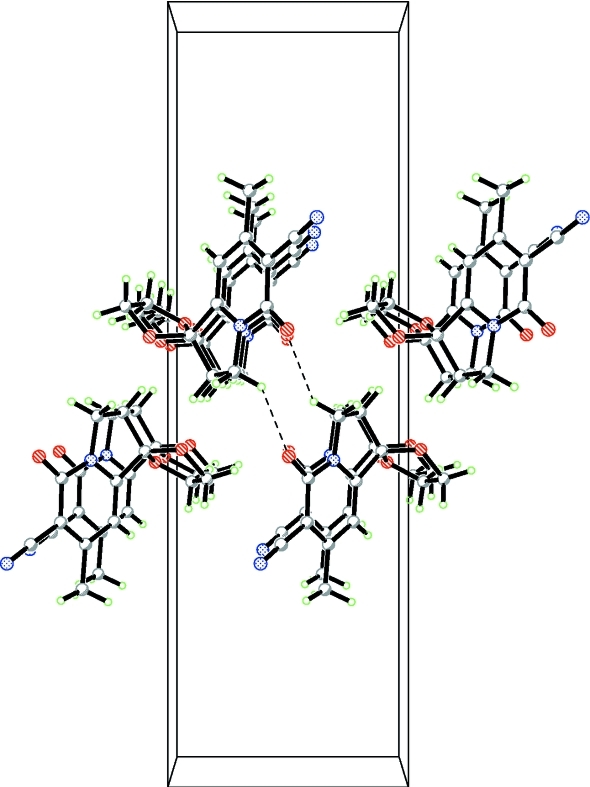
A practical packing diagram of the title compound. Hydron bonds are shown as dashed lines.

## supplementary crystallographic information

### Comment

Camptothecin(CPT), with the chemical name (*S*)-4-ethyl-4-hydroxy-1*H*-pyr- ano[3',4':6,7]indolizino[1,2-*b*]quinoline-3,14-(4*H*,12H)-dione, is a pentacyclic alkaloid. Two CPT analogues, topotecan and irinotecan, have been approved and are used in cancer chemotherapy (Takimoto & Calvo, 2008). As part of our studies into the synthesis of Camptothecin, the title compound, (I), was synthesized (Wani *et al.*, 1980). We report herein the crystal structure of the title compound.

In the molecule of the title compound, (Fig. 1), the bond lengths (Allen *et al.*, 1987) and angles are within normal ranges. In the crystal structure, C—H···O hydrogen bonds link the molecules (Fig. 2), based on the geometrically positioned H5B atom, in which they may be effective in the stabilization of the structure.

### Experimental

A mixture of 1,2,3,5-tetrahydro-7-methyl-1,5-dioxo-6- Indolizinecarbonitrile, (13.0 g,0.069 mol), ethylene glycol (210 ml), and *p*-toluenesulfonic acid (1.1 g) in toluene (1.0 *L*) was refluxed using a Dean-Stark trap for 5 h. The toluene layer was decanted and another liter of toluene was added. The reaction mixture was refluxed for an additional 5 h and the toluene layer decanted as before. After repeating this procedure two more times, the toluene layers were combined, washed with brine, dried, and evaporated to yield the crude product, which was crystallized from MeOH to give the title compound (93%) (Wani *et al.*, 1980). Colourless blocks of (I) were obtained by slow evaporation of an MeOH solution.

### Refinement

Anomalous dispersion was negligible and Friedel pairs were merged before refinement. H atoms were positioned geometrically with C—H = 0.93, 0.98 and 0.97 Å for aromatic, methine and methylene H atoms, respectively, and constrained to ride on their parent atoms, with *U*_iso_(H) = 1.2 (or 1.5 for methyl groups) times *U*_eq_(C).

### Crystal data

**Table:**

C_12_H_12_N_2_O_3_	*D*_x_ = 1.400 Mg m^−^^3^
*M**_r_* = 232.24	Mo *K*α radiation, λ = 0.71073 Å
Orthorhombic, *P**n**a*2_1_	Cell parameters from 25 reflections
*a* = 7.9460 (16) Å	θ = 9–12°
*b* = 25.945 (5) Å	µ = 0.10 mm^−^^1^
*c* = 5.3430 (11) Å	*T* = 293 K
*V* = 1101.5 (4) Å^3^	Block, colorless
*Z* = 4	0.30 × 0.10 × 0.10 mm
*F*(000) = 488	

### Data collection

**Table:**

Enraf–Nonius CAD-4 diffractometer	821 reflections with *I* > 2σ(*I*)
Radiation source: fine-focus sealed tube	*R*_int_ = 0.035
graphite	θ_max_ = 25.3°, θ_min_ = 1.6°
ω/2θ scans	*h* = 0→9
Absorption correction: ψ scan (North *et al.*, 1968)	*k* = −31→31
*T*_min_ = 0.970, *T*_max_ = 0.990	*l* = 0→6
2217 measured reflections	3 standard reflections every 200 reflections
1123 independent reflections	intensity decay: 1%

### Refinement

**Table:**

Refinement on *F*^2^	Secondary atom site location: difference Fourier map
Least-squares matrix: full	Hydrogen site location: inferred from neighbouring sites
*R*[*F*^2^ > 2σ(*F*^2^)] = 0.046	H-atom parameters constrained
*wR*(*F*^2^) = 0.112	*w* = 1/[σ^2^(*F*_o_^2^) + (0.063*P*)^2^] where *P* = (*F*_o_^2^ + 2*F*_c_^2^)/3
*S* = 1.00	(Δ/σ)_max_ < 0.001
1123 reflections	Δρ_max_ = 0.20 e Å^−^^3^
155 parameters	Δρ_min_ = −0.20 e Å^−^^3^
1 restraint	Extinction correction: *SHELXL97* (Sheldrick, 2008), Fc^*^=kFc[1+0.001xFc^2^λ^3^/sin(2θ)]^-1/4^
Primary atom site location: structure-invariant direct methods	Extinction coefficient: 0.047 (6)

### Special details

**Table:**

Geometry. All e.s.d.'s (except the e.s.d. in the dihedral angle between two l.s. planes) are estimated using the full covariance matrix. The cell e.s.d.'s are taken into account individually in the estimation of e.s.d.'s in distances, angles and torsion angles; correlations between e.s.d.'s in cell parameters are only used when they are defined by crystal symmetry. An approximate (isotropic) treatment of cell e.s.d.'s is used for estimating e.s.d.'s involving l.s. planes.
Refinement. Refinement of *F*^2^ against ALL reflections. The weighted *R*-factor *wR* and goodness of fit *S* are based on *F*^2^, conventional *R*-factors *R* are based on *F*, with *F* set to zero for negative *F*^2^. The threshold expression of *F*^2^ > σ(*F*^2^) is used only for calculating *R*-factors(gt) *etc*. and is not relevant to the choice of reflections for refinement. *R*-factors based on *F*^2^ are statistically about twice as large as those based on *F*, and *R*- factors based on ALL data will be even larger.

### Fractional atomic coordinates and isotropic or equivalent isotropic displacement parameters (Å^2^)

**Table:**

	*x*	*y*	*z*	*U*_iso_*/*U*_eq_	
N1	0.6878 (4)	0.41701 (10)	0.1075 (7)	0.0350 (8)	
O1	1.0548 (3)	0.42931 (10)	−0.0779 (6)	0.0432 (7)	
C1	1.1570 (5)	0.39063 (18)	−0.1891 (10)	0.0523 (12)	
H1A	1.1499	0.3586	−0.0963	0.063*	
H1B	1.2737	0.4015	−0.1965	0.063*	
O2	0.9270 (3)	0.41052 (10)	−0.4426 (6)	0.0464 (8)	
C2	1.0842 (5)	0.38466 (17)	−0.4486 (10)	0.0532 (12)	
H2A	1.1579	0.4001	−0.5724	0.064*	
H2B	1.0690	0.3485	−0.4892	0.064*	
N2	0.3916 (5)	0.29581 (15)	0.6093 (9)	0.0675 (12)	
O3	0.5035 (3)	0.42382 (11)	0.4332 (7)	0.0551 (8)	
C3	0.8959 (5)	0.42629 (15)	−0.1938 (8)	0.0382 (9)	
C4	0.7840 (4)	0.38909 (13)	−0.0515 (8)	0.0330 (8)	
C5	0.7124 (5)	0.47294 (14)	0.0789 (9)	0.0435 (10)	
H5A	0.7800	0.4868	0.2142	0.052*	
H5B	0.6055	0.4910	0.0735	0.052*	
C6	0.8043 (5)	0.47679 (15)	−0.1705 (9)	0.0465 (11)	
H6A	0.7254	0.4813	−0.3072	0.056*	
H6B	0.8828	0.5054	−0.1701	0.056*	
C7	0.7736 (4)	0.33684 (13)	−0.0581 (8)	0.0378 (9)	
H7A	0.8364	0.3182	−0.1736	0.045*	
C8	0.6665 (4)	0.31108 (13)	0.1120 (8)	0.0361 (9)	
C9	0.5774 (4)	0.34061 (15)	0.2809 (8)	0.0384 (9)	
C10	0.5825 (5)	0.39580 (15)	0.2880 (8)	0.0411 (10)	
C11	0.6517 (5)	0.25394 (13)	0.1031 (9)	0.0484 (11)	
H11A	0.5747	0.2426	0.2301	0.073*	
H11B	0.7602	0.2387	0.1318	0.073*	
H11C	0.6107	0.2436	−0.0583	0.073*	
C12	0.4728 (5)	0.31630 (16)	0.4644 (10)	0.0451 (10)	

### Atomic displacement parameters (Å^2^)

**Table:**

	*U*^11^	*U*^22^	*U*^33^	*U*^12^	*U*^13^	*U*^23^
N1	0.0381 (16)	0.0350 (16)	0.0320 (18)	0.0017 (13)	0.0036 (16)	−0.0016 (17)
O1	0.0423 (14)	0.0509 (14)	0.0365 (15)	−0.0026 (13)	0.0029 (16)	−0.0031 (14)
C1	0.049 (2)	0.059 (3)	0.049 (3)	0.006 (2)	0.006 (3)	0.006 (3)
O2	0.0522 (16)	0.0618 (18)	0.0252 (14)	0.0076 (14)	0.0057 (15)	0.0018 (14)
C2	0.061 (3)	0.057 (2)	0.042 (2)	0.009 (2)	0.018 (2)	0.000 (2)
N2	0.065 (2)	0.081 (3)	0.056 (3)	−0.017 (2)	0.022 (3)	0.002 (2)
O3	0.0598 (18)	0.0526 (16)	0.0528 (18)	0.0025 (14)	0.0263 (18)	−0.0127 (17)
C3	0.043 (2)	0.044 (2)	0.027 (2)	0.0000 (17)	0.003 (2)	−0.0032 (19)
C4	0.0313 (18)	0.0400 (19)	0.0276 (18)	0.0043 (16)	0.0004 (19)	−0.003 (2)
C5	0.054 (2)	0.038 (2)	0.038 (2)	0.0027 (18)	0.009 (2)	0.000 (2)
C6	0.055 (2)	0.042 (2)	0.042 (2)	0.003 (2)	0.006 (2)	0.010 (2)
C7	0.040 (2)	0.039 (2)	0.034 (2)	0.0055 (16)	0.009 (2)	−0.004 (2)
C8	0.0340 (18)	0.041 (2)	0.033 (2)	0.0011 (16)	0.001 (2)	−0.002 (2)
C9	0.037 (2)	0.043 (2)	0.034 (2)	−0.0003 (18)	0.003 (2)	0.001 (2)
C10	0.039 (2)	0.046 (2)	0.038 (2)	0.0061 (18)	0.001 (2)	−0.004 (2)
C11	0.053 (2)	0.042 (2)	0.050 (3)	−0.0047 (17)	0.008 (3)	0.004 (3)
C12	0.041 (2)	0.052 (2)	0.043 (3)	−0.0045 (19)	0.005 (2)	0.000 (2)

### Geometric parameters (Å, °)

**Table:**

N1—C4	1.353 (5)	C4—C7	1.359 (5)
N1—C10	1.390 (5)	C5—C6	1.523 (6)
N1—C5	1.472 (4)	C5—H5A	0.9700
O1—C3	1.408 (5)	C5—H5B	0.9700
O1—C1	1.421 (5)	C6—H6A	0.9700
C1—C2	1.510 (7)	C6—H6B	0.9700
C1—H1A	0.9700	C7—C8	1.413 (5)
C1—H1B	0.9700	C7—H7A	0.9300
O2—C3	1.413 (5)	C8—C9	1.379 (6)
O2—C2	1.419 (5)	C8—C11	1.488 (5)
C2—H2A	0.9700	C9—C12	1.432 (6)
C2—H2B	0.9700	C9—C10	1.433 (5)
N2—C12	1.140 (5)	C11—H11A	0.9600
O3—C10	1.235 (5)	C11—H11B	0.9600
C3—C6	1.504 (6)	C11—H11C	0.9600
C3—C4	1.517 (6)		
			
C4—N1—C10	124.3 (3)	N1—C5—H5B	111.2
C4—N1—C5	112.8 (3)	C6—C5—H5B	111.2
C10—N1—C5	122.8 (3)	H5A—C5—H5B	109.1
C3—O1—C1	106.8 (3)	C3—C6—C5	104.3 (3)
O1—C1—C2	103.7 (4)	C3—C6—H6A	110.9
O1—C1—H1A	111.0	C5—C6—H6A	110.9
C2—C1—H1A	111.0	C3—C6—H6B	110.9
O1—C1—H1B	111.0	C5—C6—H6B	110.9
C2—C1—H1B	111.0	H6A—C6—H6B	108.9
H1A—C1—H1B	109.0	C4—C7—C8	119.5 (4)
C3—O2—C2	108.2 (3)	C4—C7—H7A	120.3
O2—C2—C1	105.6 (4)	C8—C7—H7A	120.3
O2—C2—H2A	110.6	C9—C8—C7	117.9 (3)
C1—C2—H2A	110.6	C9—C8—C11	122.3 (4)
O2—C2—H2B	110.6	C7—C8—C11	119.9 (4)
C1—C2—H2B	110.6	C8—C9—C12	120.1 (3)
H2A—C2—H2B	108.8	C8—C9—C10	123.9 (4)
O1—C3—O2	105.9 (3)	C12—C9—C10	116.0 (4)
O1—C3—C6	110.4 (3)	O3—C10—N1	120.6 (3)
O2—C3—C6	114.5 (4)	O3—C10—C9	126.2 (4)
O1—C3—C4	109.9 (3)	N1—C10—C9	113.2 (3)
O2—C3—C4	112.9 (3)	C8—C11—H11A	109.5
C6—C3—C4	103.2 (3)	C8—C11—H11B	109.5
N1—C4—C7	121.1 (4)	H11A—C11—H11B	109.5
N1—C4—C3	107.8 (3)	C8—C11—H11C	109.5
C7—C4—C3	131.1 (4)	H11A—C11—H11C	109.5
N1—C5—C6	102.7 (3)	H11B—C11—H11C	109.5
N1—C5—H5A	111.2	N2—C12—C9	178.3 (5)
C6—C5—H5A	111.2		
			
C3—O1—C1—C2	28.0 (4)	O1—C3—C6—C5	−87.6 (4)
C3—O2—C2—C1	−5.9 (5)	O2—C3—C6—C5	153.0 (3)
O1—C1—C2—O2	−13.4 (5)	C4—C3—C6—C5	29.8 (4)
C1—O1—C3—O2	−32.5 (4)	N1—C5—C6—C3	−27.3 (4)
C1—O1—C3—C6	−157.0 (4)	N1—C4—C7—C8	−3.0 (6)
C1—O1—C3—C4	89.8 (4)	C3—C4—C7—C8	174.6 (4)
C2—O2—C3—O1	23.4 (4)	C4—C7—C8—C9	−0.6 (6)
C2—O2—C3—C6	145.3 (3)	C4—C7—C8—C11	178.8 (4)
C2—O2—C3—C4	−96.9 (4)	C7—C8—C9—C12	−177.3 (4)
C10—N1—C4—C7	5.3 (6)	C11—C8—C9—C12	3.3 (6)
C5—N1—C4—C7	−177.6 (4)	C7—C8—C9—C10	2.3 (6)
C10—N1—C4—C3	−172.8 (3)	C11—C8—C9—C10	−177.1 (4)
C5—N1—C4—C3	4.3 (5)	C4—N1—C10—O3	177.2 (4)
O1—C3—C4—N1	96.2 (4)	C5—N1—C10—O3	0.4 (6)
O2—C3—C4—N1	−145.8 (3)	C4—N1—C10—C9	−3.5 (6)
C6—C3—C4—N1	−21.6 (4)	C5—N1—C10—C9	179.8 (3)
O1—C3—C4—C7	−81.7 (5)	C8—C9—C10—O3	179.0 (4)
O2—C3—C4—C7	36.3 (6)	C12—C9—C10—O3	−1.5 (6)
C6—C3—C4—C7	160.5 (4)	C8—C9—C10—N1	−0.4 (6)
C4—N1—C5—C6	14.6 (4)	C12—C9—C10—N1	179.2 (3)
C10—N1—C5—C6	−168.3 (3)		

### Hydrogen-bond geometry (Å, °)

**Table:**

*D*—H···*A*	*D*—H	H···*A*	*D*···*A*	*D*—H···*A*
C5—H5B···O3^i^	0.97	2.49	3.275 (5)	138

Symmetry codes: (i) −*x*+1, −*y*+1, *z*−1/2.
